# Evaluating the performance of polygenic risk profiling across diverse ancestry populations in Parkinson’s disease

**DOI:** 10.1101/2023.11.28.23299090

**Published:** 2023-11-29

**Authors:** Paula Saffie-Awad, Inas Elsayed, Arinola O. Sanyaolu, Peter Wild Crea, Artur F Schumacher Schuh, Kristin S Levine, Dan Vitale, Mathew J Korestky, Jeffrey Kim, Thiago Peixoto Leal, María Teresa Periñan, Sumit Dey, Alastair J Noyce, Armando Reyes-Palomares, Noela Rodriguez-Losada, Jia Nee Foo, Wael Mohamed, Karl Heilbron, Lucy Norcliffe-Kaufmann, the 23andMe Research Team, Mie Rizig, Njideka Okubadejo, Mike Nalls, Cornelis Blauwendraat, Andrew Singleton, Hampton Leonard, Mary B Makarious, Ignacio F. Mata, Sara Bandres-Ciga

**Affiliations:** 1. Programa de Pós-Graduação em Ciências Médicas, Universidade Federal do Rio Grande do Sul, Porto Alegre, Brazil; 2. Centro de Trastornos del Movimiento (CETRAM), Santiago, Chile; 3. Clínica Santa María, Santiago, Chile; 4. Faculty of Pharmacy, University of Gezira, Wadmadani, 20, Sudan; 5. Department of Anatomy, College of Medicine, University of Lagos, Nigeria; 6. Center for Alzheimer’s and Related Dementias (CARD), National Institute on Aging and National Institute of Neurological Disorders and Stroke, National Institutes of Health, Bethesda, MD, USA, 20814; 7. Laboratory of Neurogenetics, National Institute on Aging, National Institutes of Health, Bethesda, MD, USA; 8. Serviço de Neurologia, Hospital de Clínicas de Porto Alegre, Porto Alegre, Brazil; 9. Departamento de Farmacologia, Universidade Federal do Rio Grande do Sul, Porto Alegre, Brazil; 10. Data Tecnica International LLC, Washington, DC, USA; 11. Genomic Medicine, Lerner Research Institute, Cleveland Clinic Foundation, Cleveland, OH, USA; 12. Unidad de Trastornos del Movimiento, Servicio de Neurología y Neurofisiología Clínica, Instituto de Biomedicina de Sevilla, Hospital Universitario Virgen del Rocío/CSIC/Universidad de Sevilla, Seville, Spain; 13. Centro de Investigación Biomédica en Red sobre Enfermedades Neurodegenerativas (CIBERNED), Madrid, Spain; 14. Preventive Neurology Unit, Wolfson Institute of Population Health, Queen Mary University of London, London, UK; 15. Department of Molecular Biology and Biochemistry, Faculty of Sciences, University of Málaga, Málaga, Spain; 16. Faculty of Education Sciences, University of Málaga, Málaga, Spain; 17. Lee Kong Chian School of Medicine, Nanyang Technological University Singapore, Singapore, Singapore; 18. Laboratory of Neurogenetics, Genome Institute of Singapore, A*STAR, Singapore, Singapore; 19. Neuroscience Unit, Clinical Pharmacology Dept., Menoufiia Medical School, Egypt; 20. 23andMe, Inc., Sunnyvale, CA, USA; 21. Department of Neuromuscular Diseases, UCL Queen Square Institute of Neurology, London, WC1N 3BG, United Kingdom.; 22. Department of Medicine, College of Medicine, University of Lagos, Nigeria.; 23. German Center for Neurodegenerative Diseases (DZNE), Tübingen, Germany

## Abstract

**Objective:**

This study aims to address disparities in risk prediction by evaluating the performance of polygenic risk score (PRS) models using the 90 risk variants across 78 independent loci previously linked to Parkinson’s disease (PD) risk across seven diverse ancestry populations.

**Methods:**

We conducted a multi-stage study, testing PRS models in predicting PD status across seven different ancestries applying three approaches: 1) PRS adjusted by gender and age; 2) PRS adjusted by gender, age and principal components (PCs); and 3) PRS adjusted by gender, age and percentage of population admixture. These models were built using the largest four population-specific summary statistics of PD risk to date (base data) and individual level data obtained from the Global Parkinson’s Genetics Program (target data). We performed power calculations to estimate the minimum sample size required to conduct these analyses. A total of 91 PRS models were developed to investigate cumulative known genetic variation associated with PD risk and age of onset in a global context.

**Results:**

We observed marked heterogeneity in risk estimates across non-European ancestries, including East Asians, Central Asians, Latino/Admixed Americans, Africans, African admixed, and Ashkenazi Jewish populations. Risk allele patterns for the 90 risk variants yielded significant differences in directionality, frequency, and magnitude of effect. PRS did not improve in performance when predicting disease status using similar base and target data across multiple ancestries, demonstrating that cumulative PRS models based on current known risk are inherently biased towards European populations. We found that PRS models adjusted by percentage of admixture outperformed models that adjusted for conventional PCs in highly admixed populations. Overall, the clinical utility of our models in individually predicting PD status is limited in concordance with the estimates observed in European populations.

**Interpretation:**

This study represents the first comprehensive assessment of how PRS models predict PD risk and age at onset in a multi-ancestry fashion. Given the heterogeneity and distinct genetic architecture of PD across different populations, our assessment emphasizes the need for larger and diverse study cohorts of individual-level target data and well-powered ancestry-specific summary statistics. Our current understanding of PD status unraveled through GWAS in European populations is not generally applicable to other ancestries. Future studies should integrate clinical and *omics level data to enhance the accuracy and predictive power of PRS across diverse populations.

## Summary for Social Media

Authors’ Twitter Handles:

@ Summary:

In the Parkinson’s disease (PD) genetics field, polygenic risk assessments have traditionally been conducted on European ancestry populations, and very little is known about its applicability across diverse ancestry populations. This study aims to provide insights into the multi-ancestry performance of predictive polygenic risk score models using the 90 loci linked to PD risk in the European population. Our results show that using ancestry-specific summary statistics on the same target population does not necessarily equate to the best performance, and that PRS adjusted by percentage of population admixture is not any more effective than the conventional PRS model (baseline). Given the heterogeneity and distinct genetic architecture of PD risk across populations as well as the sample size limitation on their predictive statistical power, PRS models created from European known risk are only of limited applicability in individuals of different ancestries.

## INTRODUCTION

The heritability attributed to idiopathic Parkinson’s disease (PD) in European populations is estimated to be around 22% ^1^. Genome-wide association studies (GWAS) have been key at identifying common loci that contribute to PD risk. A total of 90 risk variants across 78 independent loci have been associated with PD risk in the European ancestry populations ^1^. More recently, large-scale efforts are focusing on increasing genetic diversity in PD studies to unravel the genetic architecture of disease across ancestries ^2–5^. The largest trans-ethnic PD GWAS meta-analysis to date performed in European, East Asian, Latino/Admixed American, and African ancestry populations identified a total of 78 loci, 12 of which had not been previously identified ^6^.

A polygenic risk score (PRS) can be generated to estimate an individual’s susceptibility to a binary outcome, exploring the cumulative estimated effect of common genetic variants on an individual’s phenotype like PD ^7,8^. However, PRS alone has not been shown to have clinical utility in predicting PD in European populations, with only 56.9% sensitivity and 63.2% specificity at best to predict disease ^9^. PRS utility improves both sensitivity (83.4%) and specificity (90.3%) to predict disease when including relevant clinical criteria such as olfactory function, family history, age, and gender ^9,10^. Similarly, the integration of environmental factors ameliorates case/control stratification ^10,11^ while the combination of multi-omics and clinical criteria in PRS models boosts prediction models across multiple diseases ^11,12^.

PRS estimates are still limited by cohort size, sparse or inconsistent clinical characteristics, and especially by a lack of diverse genetic background, as most GWAS data are only available for Europeans. Using PRS to calculate disease risk in a single population may exacerbate existing health disparities as it cannot be accurately implemented across diverse ancestries ^13,14^. To date, this limitation has been underscored by a number of studies in diseases such as coronary artery disease, type 2 diabetes, and breast and prostate cancer, where PRS models largely based on European population estimates fail to predict risk accurately in a global context ^15–17^. Therefore, more studies that investigate how genetic risk of disease varies within and between different ancestral populations are needed.

In this study, we assess differences in the power, application, and generalizability of PRS models for PD by comparing European-ancestry risk and age at onset association models across six diverse non-European ancestry populations including: East Asians (EAS), Central Asians (CAS), Latino/Admixed Americans (AMR), Africans (AFR), African admixed (AAC), and Ashkenazi Jewish (AJ) individuals. Here we apply three approaches to investigate how PRS performs in a multi-ancestry context including: 1) PRS adjusted by gender and age, 2) PRS adjusted by gender, age and principal components (PCs) and, lastly, 3) PRS adjusted by gender, age (PCs) and percentage of population admixture. Lastly, we assess and compare disease risk probabilities, magnitude of effects, sensitivity, specificity, and risk heterogeneity across ancestries.

## METHODS

### Study Participants

**Figure 1:**
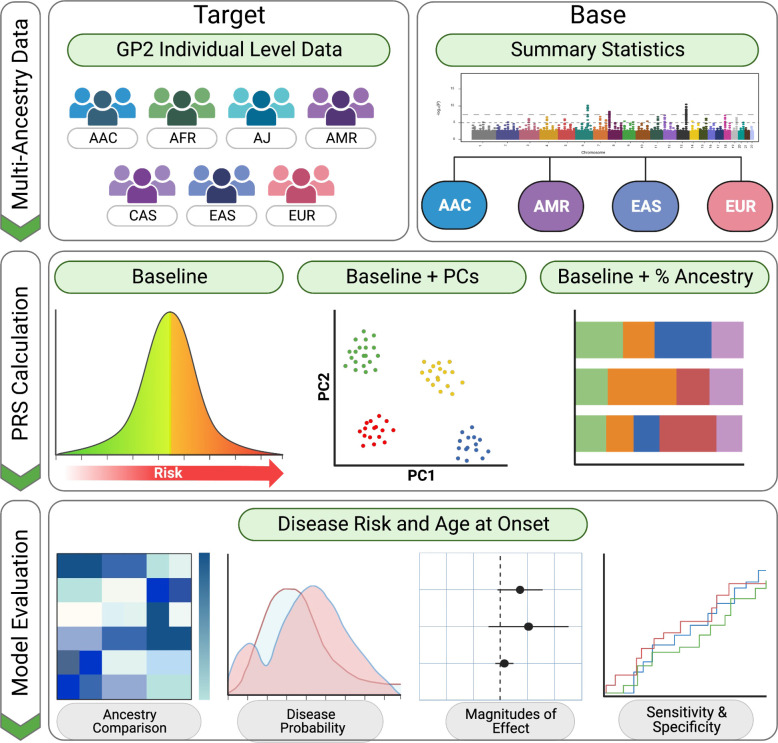
Multi-ancestry Parkinson’s disease Polygenic Risk Score (PRS) schematic workflow The figure illustrates a summarized workflow, depicting the datasets utilized, consisting of target data from seven distinct ancestry populations: African Admixed (AAC), African (AFR), Ashkenazi Jewish (AJ), Latino/Admixed American (AMR), Central Asian (CAS), East Asian (EAS), and European (EUR). The base data comprised summary statistics from four ancestries. To construct a total of 84 PRS models for PD risk and 7 PRS models for age at onset, three different approaches were implemented. The obtained results were visually presented using various plots; heatmap for ancestry comparison, density plots for disease probability, forest plots for magnitude of effect and ROC plots for sensitivity and specificity.

Our study workflow is highlighted in Figure 1. We obtained individual-level data from the Global Parkinson’s Genetics Program (GP2; DOI 10.5281/zenodo.7904832, release 5);https://gp2.org/; ^18^. This data (here referred to as target data) was used to test PRS models and comprised a total of 24,935 participants, including 12,728 individuals diagnosed with PD, 10,533 controls, and 1,674 participants diagnosed with diseases other than PD. After excluding related individuals (those who are cousins or closer) that could bias our PRS assessments and those classified as non-PD cases, our dataset comprised a total of 22,828 individuals, of which 12,551 individuals were PD cases and 10,277 individuals were controls. The following ancestries were included: African admixed, African, Ashkenazi Jewish, Latino/Admixed American, Central Asian, East Asian, and European (Supplementary Figure 1, see Methods for ancestry clustering description). Detailed demographic and clinical characteristics can be found in Table 1.

Our reference datasets (here referred to as base data) consisted of summary statistics from previously published GWAS in addition to *23andMe*.

*23andMe* participants provided informed consent and volunteered to participate in the research online, under a protocol approved by the external AAHRPP-accredited IRB, Ethical & Independent (E&I) Review Services. As of 2022, E&I Review Services is part of Salus IRB (https://www.versiticlinicaltrials.org/salusirb). The full GWAS summary statistics for the 23andMe discovery data set will be made available through 23andMe to qualified researchers under an agreement with 23andMe that protects the privacy of the 23andMe participants. Datasets will be made available at no cost for academic use. Please visit https://research.23andme.com/collaborate/#dataset-access/ for more information and to apply to access the data.

We obtained summary statistics for the European population from the largest European PD GWAS meta-analysis to date conducted by Nalls and colleagues (2019) (https://pdgenetics.org/resources). This GWAS meta-analysis included 1,456,306 individuals, of which 1,400,000 were controls, 37,688 were cases and 18,618 were proxy cases (defined as having a first degree relative with PD). African admixed summary statistics were obtained from 23andMe, which are based on 194,273 individuals including 193,985 controls and 288 cases. In order to achieve better-powered summary statistics for the East Asian population, we meta-analyzed a combination of two independent summary statistics from the largest East Asian PD GWAS meta-analysis to date ^2^ and East Asian 23andMe summary statistics, which yielded a total of 183,802 individuals, including 176,756 controls and 7,046 cases. In a similar way, we conducted GWAS meta-analysis to generate better powered Latino/Admixed American summary statistics, combining the largest Latino PD GWAS meta-analysis ^3^ with 23andMe Latino/Admixed American summary statistics. This cohort consisted of a total of 584,660 individuals, where 582,220 were controls and 2,440 PD cases. A summary of these data could be found in Supplementary Table 1.

### Sample size calculation

We conducted power calculations estimating the sample size needed to achieve an 80% power with a significance level of 0.05 in PRS models predicting disease status and age of disease onset using the methodology proposed by Dudbridge et al., ^19^. The estimations were made considering the 90 risk variants and the heritability estimates reported in Nalls et al. 2019 (defined as the percentage of the phenotype attributed to genetic variation, h^2^ = 22%) at a 0.5% general PD prevalence. The sample size required for PRS to predict disease status was 549 individuals, while for PRS predicting age at onset was 1,718. Additional details regarding the sample calculation can be found at https://github.com/DudbridgeLab/avengeme/. A total of three out of the ten ancestries (South Asian, Middle East, and Finnish ancestries) present in the GP2 cohort did not meet the minimum required sample size and therefore were excluded from further analysis.

### Target data

#### Genotype data generation and quality control

We performed genotype data generation according to standard protocols from the Global Parkinson’s Genetics Program (GP2; DOI 10.5281/zenodo.7904832, release 5; https://gp2.org/; ^18^. In summary, samples were genotyped on the NeuroBooster array (v.1.0, Illumina, San Diego, CA) that includes 1,914,935 variants encompassing ancestry informative markers, markers for identity by descent determination, and X-chromosome SNPs for sex determination. Additionally, the array includes 96,517 customized variants. Automated genotype data processing was conducted on GenoTools, a Python pipeline built for quality control and ancestry estimation of data. Additional details can be found at (https://github.com/GP2code/GenoTools).

Quality control (QC) was performed according to standard protocols. Samples with a call rate below 95%, sex mismatches, or high heterozygosity (estimated by an |F| statistics of > 0.25) were excluded from analyses. Further QC measures included the removal of SNPs with missingness above 5%, variants with significant deviations from Hardy-Weinberg Equilibrium (HWE P value < 1E-4), variants with non-random missingness (case-control status at P≤1E-4), and variants with missing data patterns (haplotype at P≤1E-4 per ancestry).

### Ancestry predictions

The samples were divided into different groups based on ancestry estimates, which involved determining the ancestral background of each sample using reference panels from the 1000 Genomes Project (https://www.internationalgenome.org/data-portal/data-collection/phase-1) ^20^, Human Genome Diversity Project ^21^, and an Ashkenazi Jewish population dataset ^22^. Our reference panel, at time of writing (July 2023), consists of 703 African, 601 South Asian, 585 East Asian, 534 European, 490 Latin American, 471 Ashkenazi Jewish, 190 African admixed, 183 Central Asian, 152 Middle Eastern, and 99 Finish individuals. We refined the reference panel by excluding palindromic SNPs (AT or TA or GC or CG). Additionally, SNPs within the reference panel underwent further filtering to exclude variants with a minor allele frequency (MAF) lower than 0.05, genotyping call rate less than 0.99, and Hardy-Weinberg equilibrium (HWE) p-value less than 1E-4. Variants that overlapped between the reference panel SNP set and the samples of interest were specifically extracted. In total, 39,302 variants were used for ancestry estimations. In cases where genotypes were missing, imputation was performed by utilizing the mean value of that variant from the reference panel.

To assess the performance of the ancestry estimation, an 80/20 train/test split was applied to the reference panel samples. PCs were then calculated using the overlapping SNPs. Transforming the PCs through UMAP enabled the representation of global genetic population substructure and stochastic variation. Training a linear support vector classifier on the UMAP transformations of the PCs achieved consistent predictions with balanced accuracies exceeding 0.95, as determined by testing the classifier on the reference panel’s test data through 5-fold cross-validation. These classifier models were subsequently applied to the GP2 data to generate ancestry estimates for all datasets. For detailed insights into the cloud-based and scalable pipeline used for genotype calling, QC, and ancestry estimation, please refer to the GenoTools GitHub repository (https://github.com/GP2code/GenoTools). Following ancestry estimation, we excluded duplicated or monozygotic twin samples (KING coefficient > 0.354), and those with second-degree or closer relatedness (KING coefficient > 0.0884). PCs that were used as covariates in the PRS analysis were calculated separately per ancestry after initial QC and ancestry prediction were complete. Percentage of ancestry was then calculated with the supervised functionality of ADMIXTURE (v1.3.0; https://dalexander.github.io/admixture/binaries/admixture_linux-1.3.0.tar.gz), which used the labeled reference panel data as training samples to estimate the ancestry proportions of the GP2 data.

### Imputation

Variants with a minor allele frequency (MAF) of less than 0.005 and Hardy-Weinberg equilibrium (HWE) p-value less than 1E-5 were excluded before submission to the TOPMed Imputation server. The utilized TOPMed reference panel version, known as r2, encompasses genetic information from 97,256 reference samples and over 300 million genetic variants across the 22 autosomes and the X chromosome. As of October 2022, the TOPMed panel includes approximately 180,000 participants, with 29% of African, 19% of Latino/Admixed American ancestry, 8% of Asian ancestry, and 40% of European ancestry (https://topmed.nhlbi.nih.gov/). Further details about the TOPMed Study ^23^, Imputation Server ^24^, and Minimac Imputation ^25^ can be accessed at https://imputation.biodatacatalyst.nhlbi.nih.gov. Following imputation, the resulting files underwent pruning based on a minor allele count (MAC) threshold of 10 and an imputation Rsq value of 0.3.

### Base data

#### Ancestry-specific summary statistics generation

A comprehensive explanation of each step to generate 23andMe summary statistics can be found elsewhere ^6^. Briefly, the 23andMe data generation process could be summarized in the following steps. After genotyping of 23andMe participants was completed, an ancestry classifier algorithm was used to determine participant ancestries based on local ancestry and reference populations. Next, phasing was performed to reconstruct haplotypes using genotyping platform-specific panels followed by imputation of missing genotypes, expanding the variant dataset using two independent reference panels. Related individuals were then excluded using a segmental identity-by-descent estimation algorithm to ensure unrelated participants. Finally, a GWAS analysis adjusted by covariates age, sex, and principal components was conducted followed by GWAS QC measures to flag potential issues with SNPs, ensuring data integrity.

For a detailed description of the methods used to generate East Asian summary statistics, refer to the study by Foo et al. ^2^ Similarly, detailed information of the Latino/Admixed American summary statistics can be found in Loesch et al. ^3^. The GWAS meta-analysis of each population was carried out using fixed effects based on beta and SE values for the 90 risk variants. This meta-analysis was conducted utilizing the METAL package, which is accessible at https://genome.sph.umich.edu/wiki/METAL_Documentation.

### Polygenic risk score calculation

For PRS calculations, we extracted the 90 risk predictors previously linked to PD risk in European populations ^1^ from GP2 individual level data (https://gp2.org/) ^18^. The risk predictors were weighted by summary statistics magnitude of effects, giving greater weight to alleles with higher risk estimates (Figure 1). Logistic or linear regression analysis was employed to predict PD status and age of onset, respectively. To assess the predictive ability of the PRS across different populations, three distinct analyses were conducted. First, PRS analyses were performed adjusting by gender and age. Then, an additional approach was conducted adjusting by age, gender and PCs to account for population substructure. Finally, a third and novel approach was applied adjusting by age, gender and percentage of ancestry admixture. These three approaches were performed across each of the seven GP2 ancestry populations (target datasets) using the four different population-specific summary statistics individually (base datasets), totaling 84 PRS models predicting disease risk and 7 PRS models predicting age at disease onset (Figure 1). The results were visualized through heatmaps for ancestry comparisons, density plots for disease probabilities, forest plots for magnitude of effects comparison, area under the curve (AUC) and receiver operating characteristic curve (ROC) assessments for sensitivity and specificity of the models. Finally, UpSet visualizations were used to display heterogeneity estimated across known loci and multiple ancestries.

## RESULTS

### Risk estimates show expected high levels of heterogeneity in predicting disease status across diverse ancestry populations

**Figure 2:**
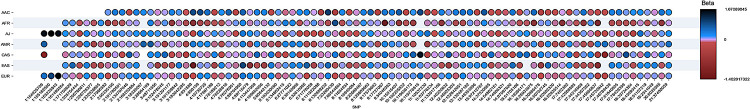
Upset plot showing risk heterogeneity across multiple ancestries. The 90 risk variants are represented in this plot in a granular way. The Y axis represents each ancestry populations and the X axis the 90 risk variants. The color bar shows the magnitude of effects as log of the odd ratio (beta value) and directionality, with red color denoting negative directionality, and purple and blue colors denoting positive directionality.

We used individual-level data from seven ancestry populations (target data) to examine risk allele patterns across the 90 risk variants (Figure 2, Supplementary Table 2). We found significant heterogeneity among these predictors when standardizing the effect allele for each estimate. When we looked at the risk patterns across different populations, we observed differences in directionality, frequency and magnitude of effect (Figure 2, Supplementary Table 3). These findings confirmed that our current understanding of PD risk is biased toward Europeans, as the 90 risk alleles assessed in the present work were originally discovered in European GWAS, leaving much genetic variability to be uncovered. For example, the *GBA1* risk variants (*GBA1*-E326K and *GBA1*-N370S) were absent when exploring the *GBA1* locus in African and African admixed ancestries due to allele frequencies and population-specific risk. African and African admixed populations rarely harbor *GBA1*-E326K and *GBA1*-N370S mutations ^4^. We envisage that *GBA1* is not the only example where differences in the genetic architecture at the locus level exist.

### Polygenic risk scores do not show higher performance in predicting disease status when using similar base and target data across multiple ancestries.

To evaluate the utility of PRS to predict disease status, we applied three regression analysis models; 1) Baseline PRS analysis (which is only adjusted by gender and age), 2) PRS adjusted by gender, age, and PCs, and 3) PRS adjusted by gender, age and percentage of admixture (Tables 2a, b, c & 3, Figure 1). PRS models built using European summary statistics (base data) showed the largest number of risk predictors retrieved across the seven studied ancestries (ranging from 83–90 SNPs) (Supplementary Table 2, Figure 2). Nevertheless, when using East Asian base data (Foo et al., 2020 – 23andMe GWAS meta-analysis), our PRS models showed limited coverage with the least number of retrieved risk predictors (ranging from 60–64) (Supplementary Table 2, Figure 2). The genetic structure across populations is different and thus so are variant imputation and allele frequencies.

**Figure 3:**
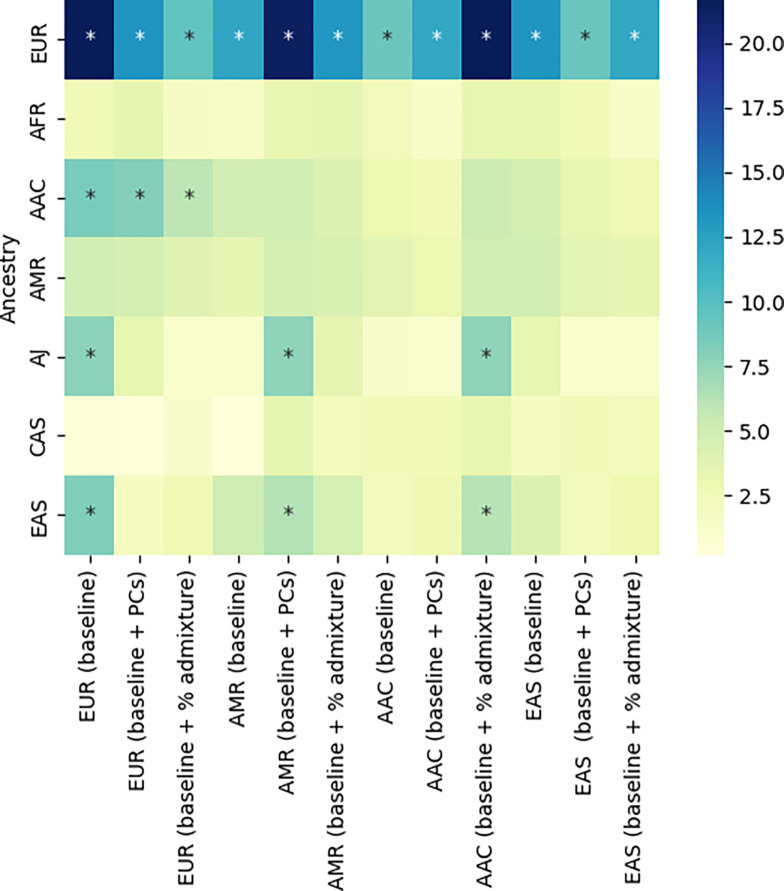
PRS performance for predicting disease status The Y axis represents individual level data, and the X axis represents the three different PRS approaches per population-specific summary statistics. The color bar indicate the magnitude of effect as log of the odds ratio (beta value). The darker the color is the larger the magnitude of effect. The asterisks indicate statistical significance of P value.

Generally, PRS models across the seven target ancestries performed better when built based on European population base data as compared to other population-specific summary statistics based PRS models (Figure 3, Table 2a, Supplementary Table 2). PRS effect sizes are summarized as follows when using European base data on; European target data (positive control);(Beta=0.52, SE =0.02), African admixed target data; (Beta=0.67, SE=0.08), Ashkenazi Jewish target data;(Beta=0.58, SE=0.08), and Latino/Admixed American target data;(Beta=0.55, SE =0.11), which all harbor certain levels of European ancestry (Supplementary Figures 4a, c, d). PRS performed poorly in the African (Beta=0.24, SE= 0.10) and Central Asian (Beta=0.06, SE=0.11) target datasets; likely reflecting the genetic heterogeneity between African and European ancestries (Supplementary Figures 4b & e, Supplementary Figure 1, Supplementary Table 2) and also the limited sample size in the Central Asian population.

PRS models built using African admixed and Latino/Admixed American base data performed poorly in all studied ancestries, even when using the corresponding ancestry target data: African admixed base data on African ancestry target data; (Beta=0.15, SE=0.1), and Latino/Admixed American base data on Latino/Admixed American ancestry target data; (Beta=0.35, SE=0.10) (Figure 3, Table 2a). This indicates that PRS models based on similar base and target data do not necessarily perform better on the specific target population; an observation that could be explained first by the limited sample size from both population-specific base and target data, and second by the distinct genetic architecture of PD risk across populations that could limit the efficiency of our PRS models built on 90 predictors nominated from European based GWASes (Table 1, Supplementary Table 1, Supplementary Table 2a).

### Polygenic risk score performance across multiple ancestries shows limited clinical utility.

Next we assessed the clinical utility of our PRS model (sensitivity and specificity in predicting PD risk) in a multi-ancestry context by estimating the ROC curve of a PRS and then calculating the AUC. Generally, the European, Ashkenazi Jewish, African admixed, and Latino/Admixed American populations produced better defined curves indicating better model performance compared to other ancestries (Supplementary Figures 3a and 3b, Table 4). The worst specificity and sensitivity values were found for the Central Asian population followed by the African population (Supplementary Figures 3a and 3b, Table 4). Overall, the clinical utility of our model in individually predicting PD risk is limited in concordance with the estimates observed in European populations. However, in line with previous reports in European populations, we envisage that integrating other variables, e.g. clinical, demographics and omics data, would markedly improve model’s sensitivity and specificity across multiple ancestries ^12^.

### Percentage of admixture adjusted polygenic risk score models outperform conventional principal component adjusted models in highly admixed populations.

Similar to the baseline approach, PRS models adjusted by gender, age and PCs built from European base data performed better in all the studied target data ancestries. In contrast, PRS models built from African admixed and Latino/Admixed American base data were less promising even on the same target population after adjusting by PCs (Tables 2a, b, Figure 3). In addition, we report limited variation in performance of the PRS model adjusted by gender, age and percentage of admixture in comparison with the PRS model adjusted by gender, age and PCs across marginally admixed populations. This novel approach only performed better than the conventional PRS model adjusted by gender, age and per specific-ancestry PCs in the African admixed and Latino/Admixed American populations (Tables 2b, c, Figure 3). Such enhancement in model performance attributed to the adjustment against population admixture improves the outcome in highly admixed populations as previously reported ^26^. This indicates that the genetic diversity within a population-represented by PCs-is well-captured in those cohorts with marginal levels of admixture. However, conventional PRS models adjusted by PCs do not optimally account for genetic substructure in highly admixed cohorts such as the African admixed and Latino/Admixed populations.

### Polygenic risk scores predicting age at onset with limited statistical power

Most of our PRS models of PD age of onset returned statistically insignificant-except for the Ashkenazi Jewish and European populations’ scores (Table 3). This was expected considering the large sample size needed to achieve desired power in this analysis as described above (see sample size calculation section of the Methods). In terms of directionality, we would expect PRS to be inversely correlated with age of onset in concordance with Nalls, 2015 ^9,27^. Accordingly, PRS is likely overall inversely correlated with PD age of onset, i.e. patients with higher genetic risk burden likely develop PD at earlier age, although statistical significance has not been achieved in most of the studied populations and further studies are needed to investigate this correlation in a multi-ancestry fashion.

## DISCUSSION

To our knowledge, this study represents the first comprehensive assessment of PRS in predicting PD risk and age at onset in a multi-ancestry context. While previous research has primarily focused on populations of European ancestry ^1,27,28^, our study expands on previous knowledge by comparing the performance of PRS across seven ancestry populations, including European, African admixed, African, Ashkenazi Jewish, Latino/Admixed American, Central Asian, and East Asian populations.

Our findings highlight the existing bias in our understanding of PD risk, which predominantly relies on European populations. By examining the 90 risk variants from the latest and largest European GWAS meta-analysis in PD across seven ancestries, we observed differences in the directionalities of these predictors in different ancestries. This indicates that risk alleles vary across populations and leaves significant genetic variability unexplored and unaccounted for. When adjusting by percentage of admixture, PRS models outperform conventional principal component adjusted models in highly admixed populations like the African admixed and Latino/Admixed American populations. The genetic heterogeneity of PD across populations highlights the need to identify additional population-specific risk variation, such as the novel intronic *GBA1* variant in the African population ^4^, *SV2C* and *WBSCR17* in East Asians, and *HEATR6* in the Chinese population ^29^ in addition to the 12 potentially novel risk loci from a recent multi-ancestry GWAS meta-analysis on PD risk ^6^.

In terms of overall performance across the seven ancestries, the best performance of PRS models - using the four summary statistics (base data) – was on our positive control, the European population. This is expected considering that we are applying a PRS model based on European GWAS nominated risk. Following the European population, Ashkenazi Jewish, African admixed, and Latino/Admixed American populations showed the largest effect sizes respectively. Not surprisingly, the models perform relatively well in these populations, which harbor certain levels of European admixture. Our results are in line with a recent study in the African admixed general population, that showed a positive correlation between PRS and percentage of European ancestry when using the 90 risk variants reported in Nalls et al., 2019 ^1,30^.

When using the same base and target specific ancestry population data, the highest PRS predictive accuracy was observed in the Latino/Admixed American population, aligning with estimations based on ancestry prediction models (Supplementary Figure 1). This is supported by the only GWAS conducted on the Latin American population, demonstrating a significant genetic resemblance to Europeans despite sample size limitations ^3^. These genetic similarities raise intriguing questions about the historical relationship between Latin Americans and Europeans, potentially stemming from shared ancestry preceding Christopher Columbus’ time. Interactions and migrations between continents may have contributed to gene flow and admixture between Latin Americans and Western Europeans, influencing the genetic landscape ^31^

However, PRS performance was more limited for African and Central Asian populations. The lower performance in Central Asian populations can be attributed to small sample sizes. The lower performance in African populations can be explained by the greater genetic diversity between African and European populations, as evidenced by the ancestry prediction models. A recent GWAS conducted on African and African admixed populations has also indicated that there is limited overlap between both genetic architectures of disease ^4^. Similar findings have been reported in prostate cancer ^32^ and breast cancer ^33^ when comparing PRS performance in Europeans vs African populations.

Several limitations should be acknowledged. Due to limited information on heritability, disease prevalence, and risk predictors for non-European ancestries, sample size power calculations were performed using current estimates from the European population as a reference. Consequently, this may result in a biased estimate regarding the sample size required to predict disease status across diverse ancestries. Additionally, the estimates of our models are influenced by the number of available SNPs in each dataset, which introduces bias. This bias arises from variations in the quality and completeness of SNP imputation across different populations, where some of them may have a larger number of imputed variants (e.g., 90 for Ashkenazi Jewish or 87 for Latino/Admixed American) compared to others (e.g., 80 for African Admixed or 83 for African). This is due to differences in variant frequencies in which common risk variation contributing to disease in Europeans is rare when assessed in a multi-ancestry context. An additional important limitation is the absence of individual-level replication datasets per ancestry. The lack of replication data hampers the robustness and generalizability of our findings across different ancestral populations. Furthermore, the scalability of our framework is hindered by the absence of accurate and well-powered ancestry-specific summary statistics for each population in our study. This is particularly challenging due to the linkage disequilibrium between SNPs from multiple ancestries, making it difficult to accurately assess specific genetic architectures of disease.

To overcome the limitations of our study, future research should prioritize larger sample sizes, replication datasets per ancestry, and improved availability of well-powered ancestry-specific summary statistics. Moreover, incorporating local ancestry information, that is the inference of the genetic ancestry of each region of each chromosome in an admixed individual ^34^. The inclusion of local ancestry can improve PRS accuracy, especially in multi-ancestry cohorts, because it allows us to use summary statistics from the ancestry PRS panel that matches with that specific region of the chromosome of the individual that we are inferring the risk, avoiding inflation/deflation because of ancestry specific risk alleles. Studying biomarker-defined PD cohorts, as opposed to those diagnosed based on clinical diagnostic criteria, is also important, as at least 5% of individuals diagnosed with PD do not demonstrate neuronal alpha-synuclein, which is required for definitive diagnosis ^35^. Additionally, applying a multi-modality machine learning approach ^12^ that combines adjusted transcriptomics, genetics, and clinical data into a predictive model, we could provide a more comprehensive understanding of PD risk and improve prediction accuracy across diverse ancestries. By utilizing machine learning algorithms such as deep learning, complex patterns and interactions that may not be evident when using individual data modalities alone can have the potential to enhance the precision and applicability of PD risk assessment models. This would lead to improved risk prediction and personalized strategies for prevention, diagnosis, and treatment for all.

In conclusion, our study contributes to a novel exploration of multi-ancestry PRS in PD. Our findings highlight the importance of larger sample sizes, replication datasets per ancestry, well-powered ancestry-specific summary statistics, and the incorporation of local ancestry information to enhance the accuracy and predictive power of multi-ancestry PRS. Furthermore, by integrating clinical and genetic data ^9^ and adopting recently published multi-modality machine learning techniques ^12^, we might uncover complex patterns and interactions not evident with conventional approaches. As more data becomes available, leveraging tools like Tractor ^36^ and PRSice ^37^ can improve scalability and determine the optimal p-value threshold for common genetic variations predisposing to PD risk in a cumulative manner. Additionally, future studies may benefit from conducting composite PRS analysis to identify optimized SNP sets across multiple ancestries with a cumulative genetic effect for more effective risk prediction. These advancements have the potential to enhance the precision and applicability of PRS analysis in PD research, leading to personalized strategies for prevention, diagnosis, and treatment.

## Supplementary Material

Supplement 1

## Data Availability

Data were obtained from the Global Parkinson’s Genetics Program (GP2) and is accessible through a partnership with the Accelerating Medicines Partnership in Parkinson’s Disease (AMP-PD) and can be requested via the website’s application process (https://www.amp-pd.org/). GWAS summary statistics from GP2’s release 5 are available for all datasets (release 5; doi:10.5281/zenodo.7904832). 23andMe summary statistics is available upon application through their website (https://research.23andme.com/dataset-access/). GenoTools (version 10; https://github.com/GP2code/GenoTools) was used for genotyping, imputation, quality control, ancestry prediction, and data processing. A secured workspace on the Terra platform was created to conduct genetic analyses using GP2 release 5 data and summary statistics (https://app.terra.bio/). Additionally, all scripts used for this study can be found in the public domain on GitHub (https://github.com/GP2code/GP2-Multiancestry-PRS; doi:10.5281/zenodo.10211779).
