# Supplementary material for: Evaluating the performance of polygenic risk profiling across diverse ancestry populations in Parkinson’s disease
Source: medRxiv. 2023 Nov 29:2023.11.28.23299090. Preprint. [Version 1] doi: 10.1101/2023.11.28.23299090 (PMC10705647; doi:10.1101/2023.11.28.23299090)
Supplement: Supplement 1 [file media-1.zip › Supplementary material/PRS supplementary figures.pdf]

Evaluating the performance of polygenic risk profiling across diverse ancestry populations in Parkinson's disease

Supplementary figures

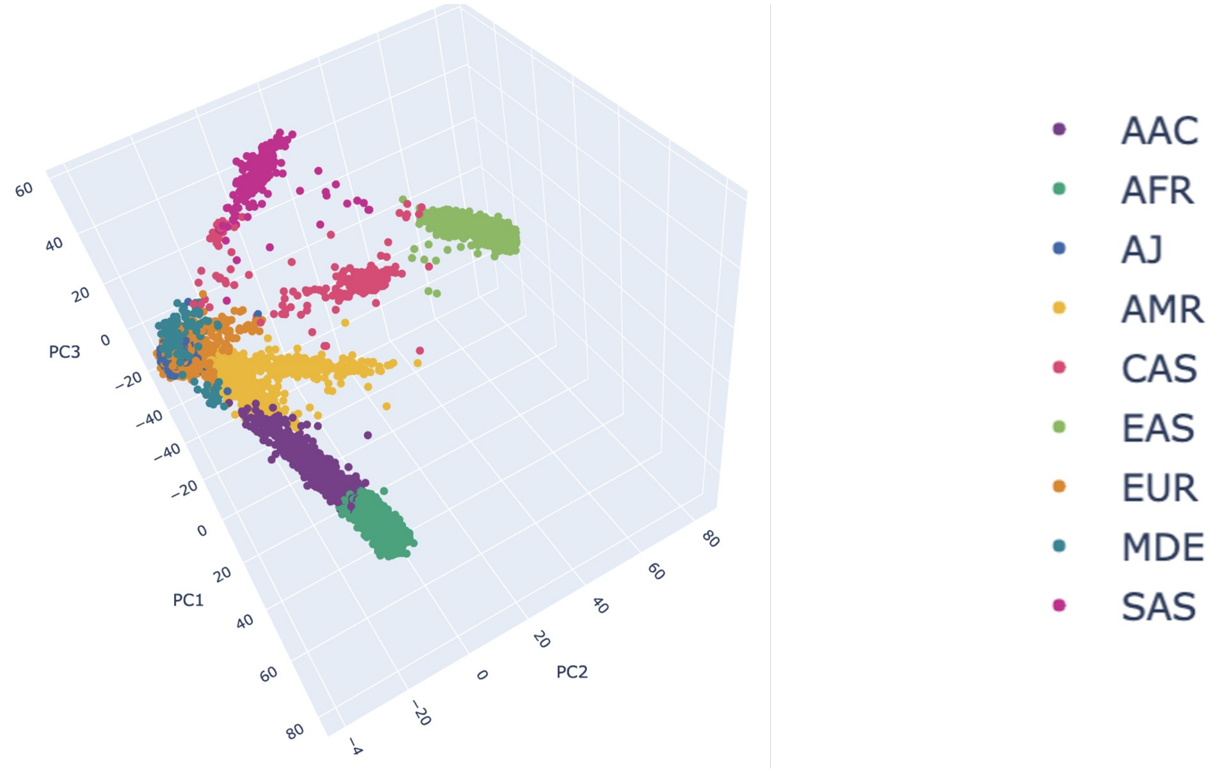

Supplementary Figure 1: Ancestry prediction model for GP2 individual level data
Utilizing 3-dimensional principal components analysis (PCA), individuals are grouped based on their genetic makeup. Each dot in the plot represents an individual, and the colors denote ancestral backgrounds, as specified in the legend: AAC (African-Admixed) in purple, AFR (African) in jade, AJ (Ashkenazi Jewish) in blue, AMR (Latino/Admixed American) in yellow, CAS (Central Asian) in pink, EAS (East Asian) in yellowish green, EUR (European) in orange, MDE (Middle East) in teal blue, and SAS (South-Asian) in fuchsia.

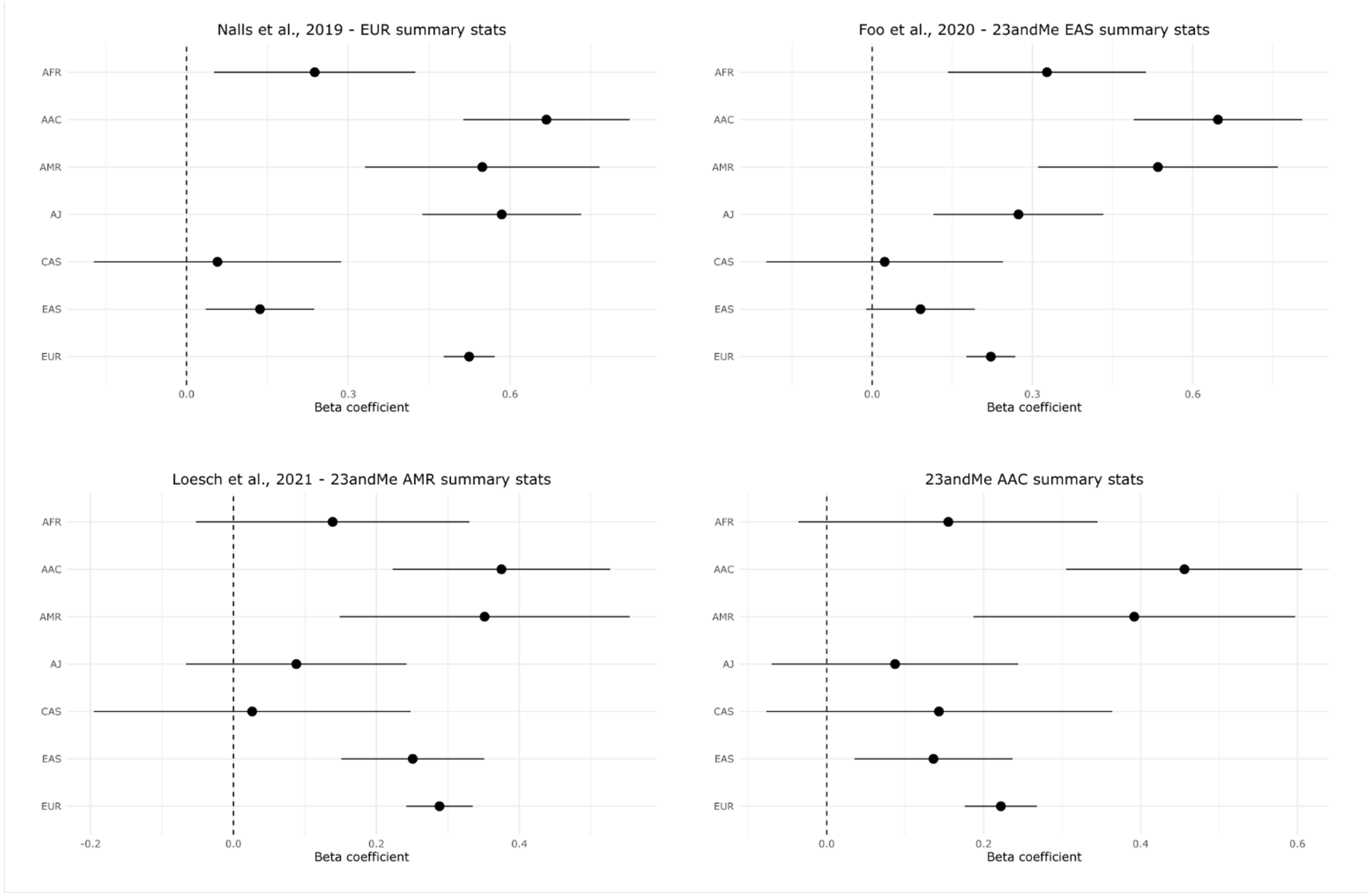

Supplementary Figure 2: Magnitude of the polygenic risk score analyses across ancestries

The forest plots visually convey the magnitude of the PRS effect in disease prediction. Each panel corresponds to the PRS using population-specific summary statistics (EUR, EAS, AMR, and AAC). The Y-axis represents individual-level data, while the X-axis illustrates the magnitude of the effect. In each panel, dots represent beta coefficients, and horizontal lines denote confidence intervals.

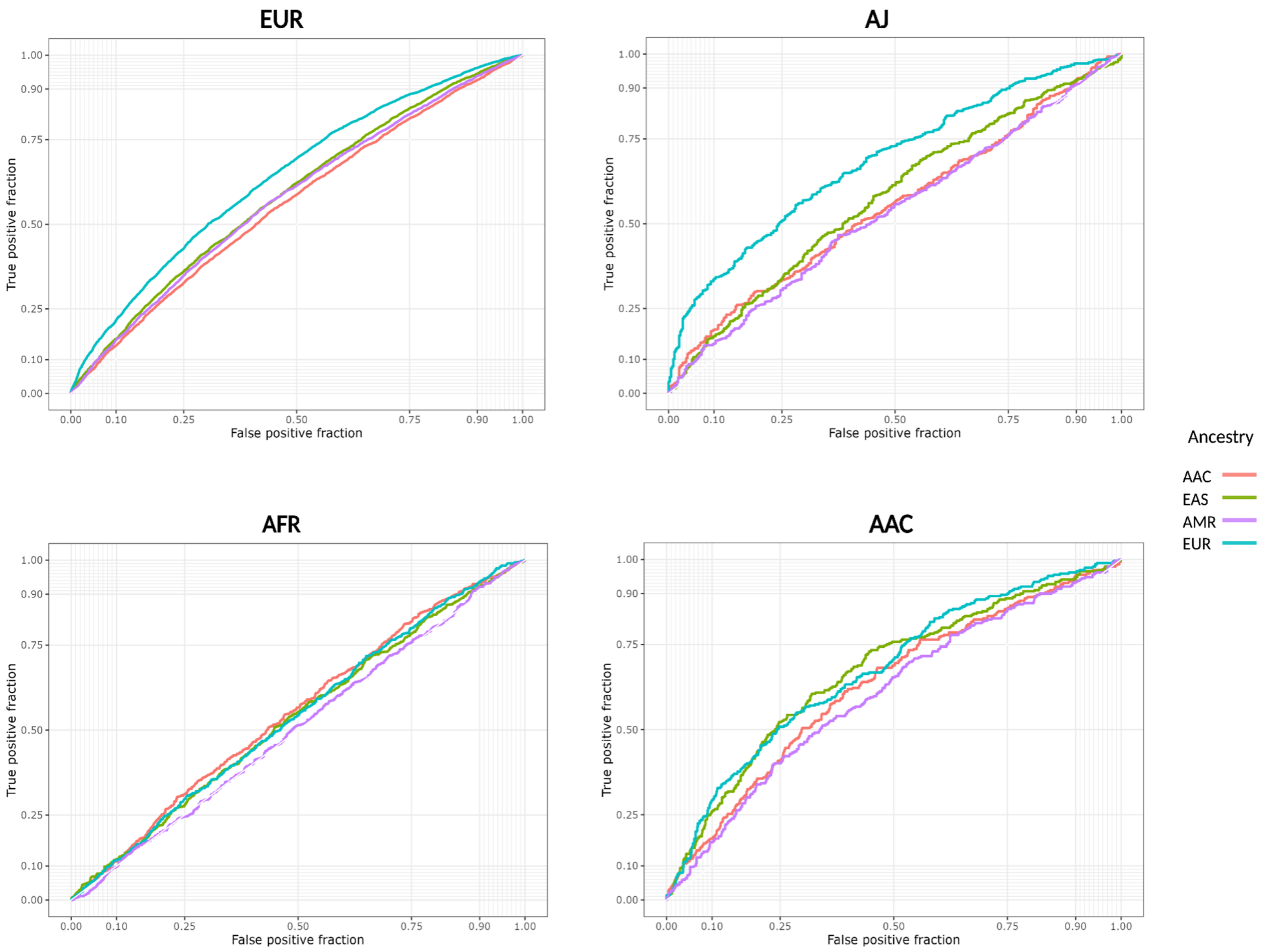

Supplementary Figure 3a: Polygenic risk score model performance evaluation

The ROC curve evaluates the PRS model's performance, depicting the true positive rate on the Y-axis against the false positive rate (1-specificity) on the X-axis. Sensitivity increases with rising Y values, while specificity decreases as X values increase. Each population-specific PRS summary statistic is represented as a curve, color-coded as follows: AAC (African-Admixed) in pink, EAS (East Asian) in yellowish green, AMR (Latino/Admixed American) in violet, and EUR (European) in light blue.

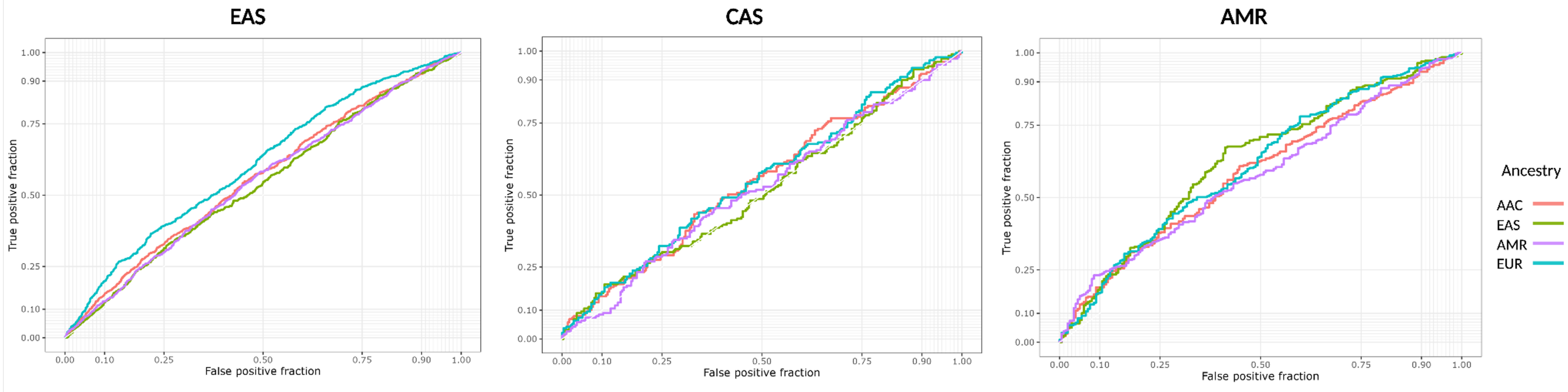

Supplementary Figure 3b: Polygenic risk score model performance evaluation

The ROC curve evaluates the PRS model's performance, depicting the true positive rate on the Y-axis against the false positive rate (1-specificity) on the X-axis. Sensitivity increases with rising Y values, while specificity decreases as X values increase. Each population-specific PRS summary statistic is represented as a curve, color-coded as follows: AAC (African-Admixed) in pink, EAS (East Asian) in yellowish green, AMR (Latino/Admixed American) in violet, and EUR (European) in light blue.

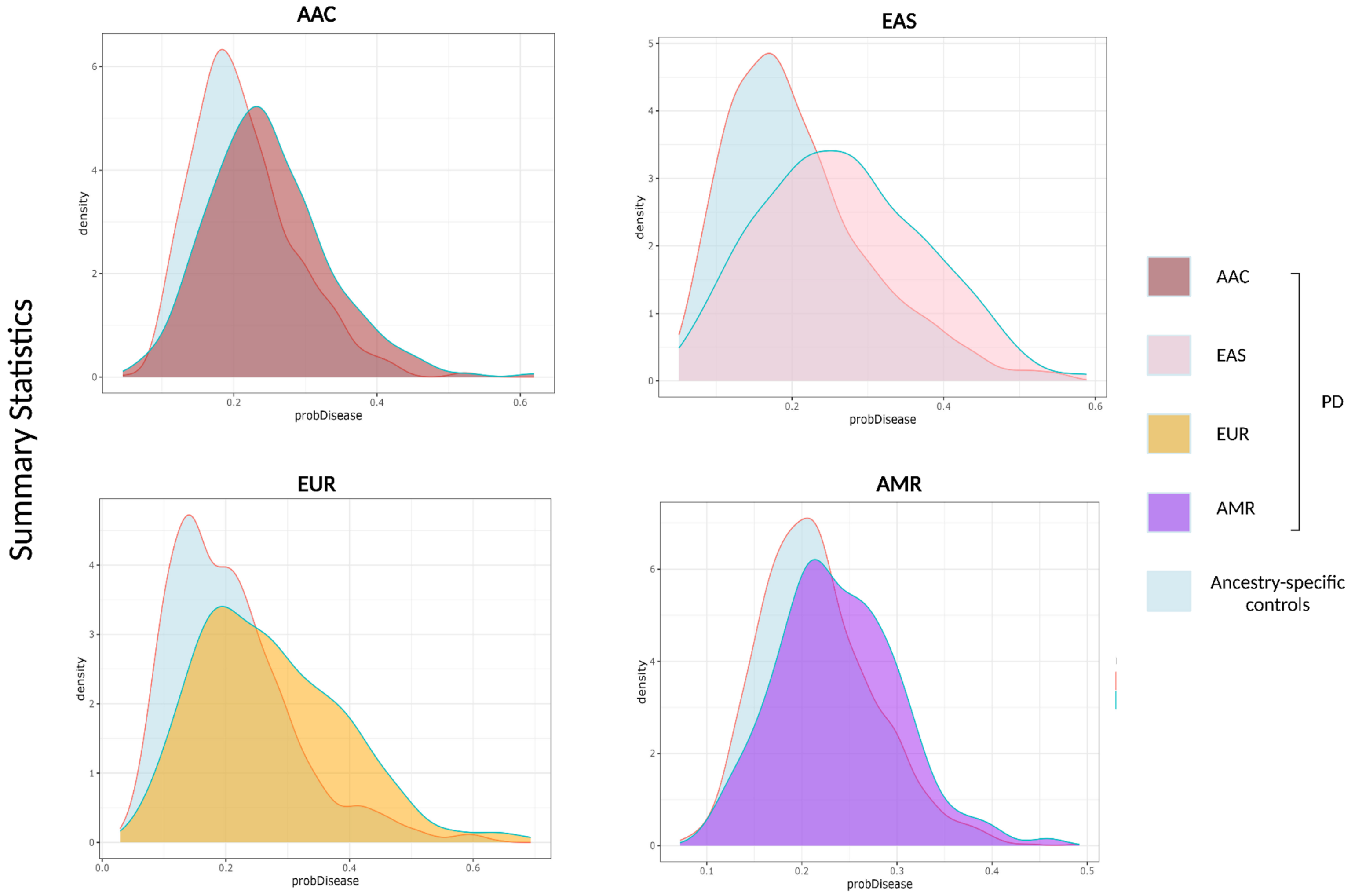

Supplementary Figure 4a: Disease probabilities in African-Admixed (AAC) individual level data
The density plot illustrates disease probabilities in AAC individual-level data, with four panels representing the performance of population specific summary statistics (EUR, AMR, EAS, and AAC) weighted PRSes. Brown, pink, yellow, and purple curves represent PD cases (AAC, EAS, EUR, and AMR, respectively), while ancestry-specific controls are depicted in light blue.

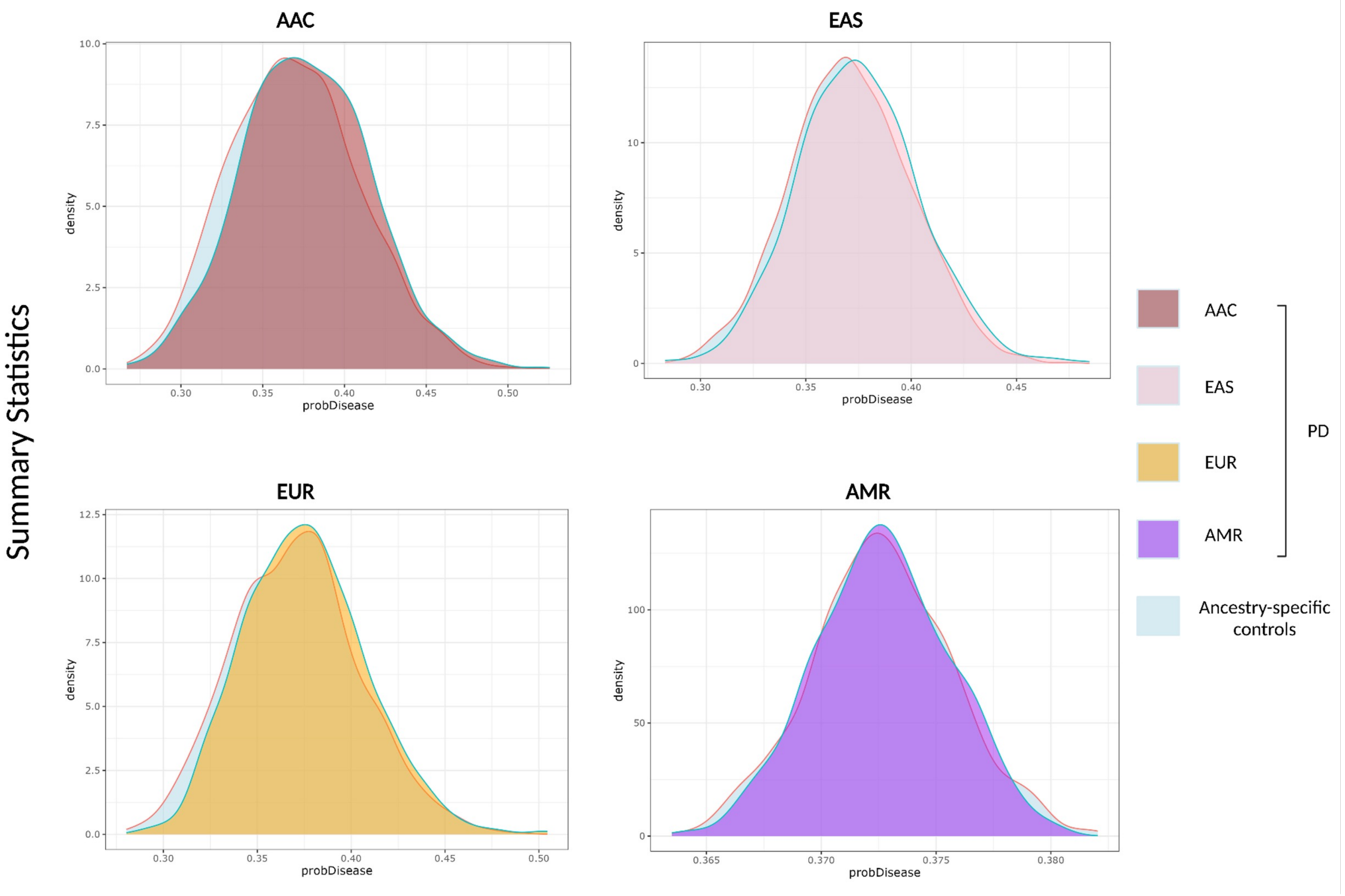

Supplementary Figure 4b: Disease probabilities in African (AFR) individual level data

The density plot illustrates disease probabilities in AAC individual-level data, with four panels representing the performance of population specific summary statistics (EUR, AMR, EAS, and AAC) weighted PRSes. Brown, pink, yellow, and purple curves represent PD cases (AAC, EAS, EUR, and AMR, respectively), while ancestry-specific controls are depicted in light blue.

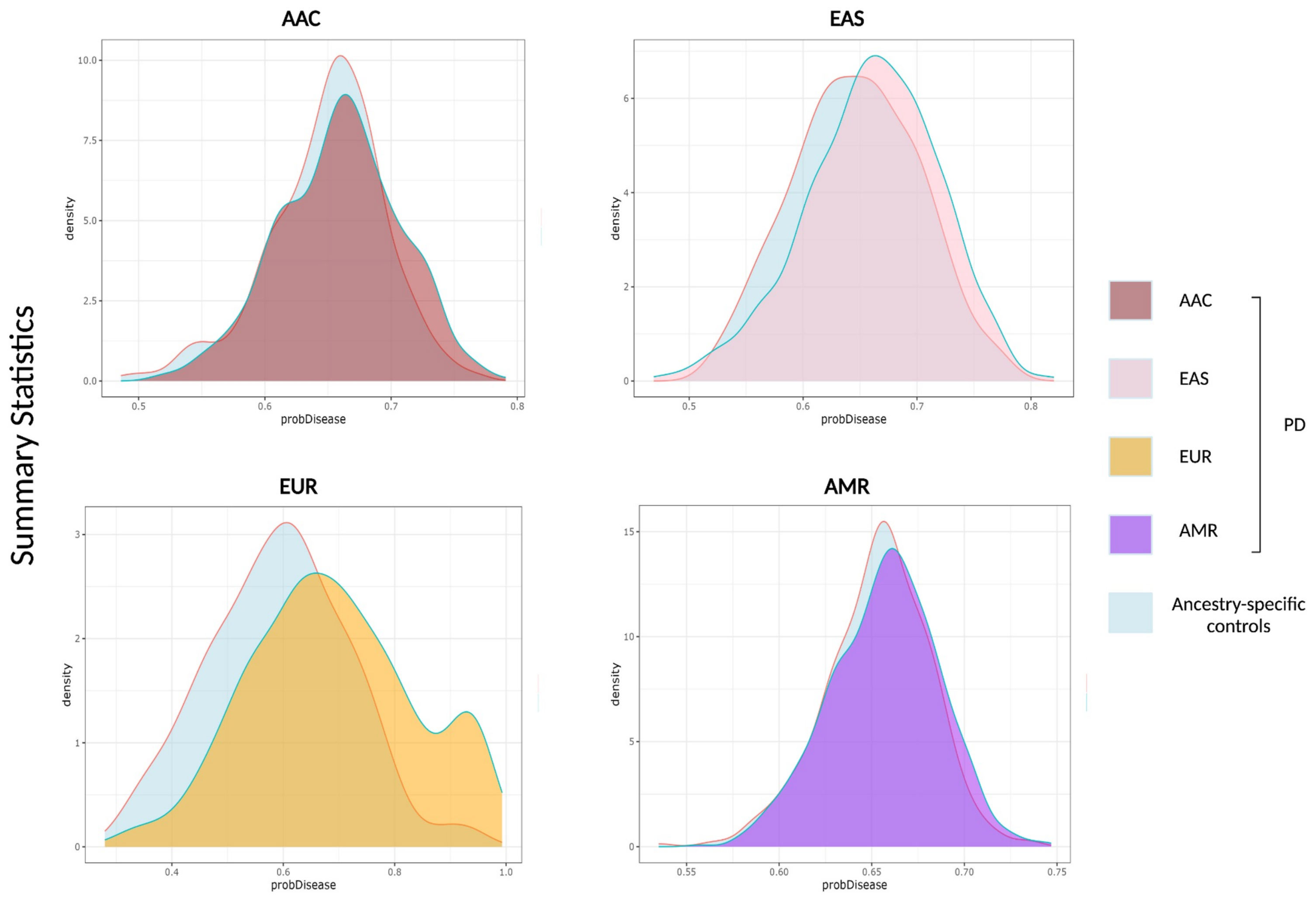

Supplementary Figure 4c: Disease probabilities in Ashkenazi Jewish (AJ) individual level data
The density plot illustrates disease probabilities in AAC individual-level data, with four panels representing the performance of population specific summary statistics (EUR, AMR, EAS, and AAC) weighted PRSes. Brown, pink, yellow, and purple curves represent PD cases (AAC, EAS, EUR, and AMR, respectively), while ancestry-specific controls are depicted in light blue.

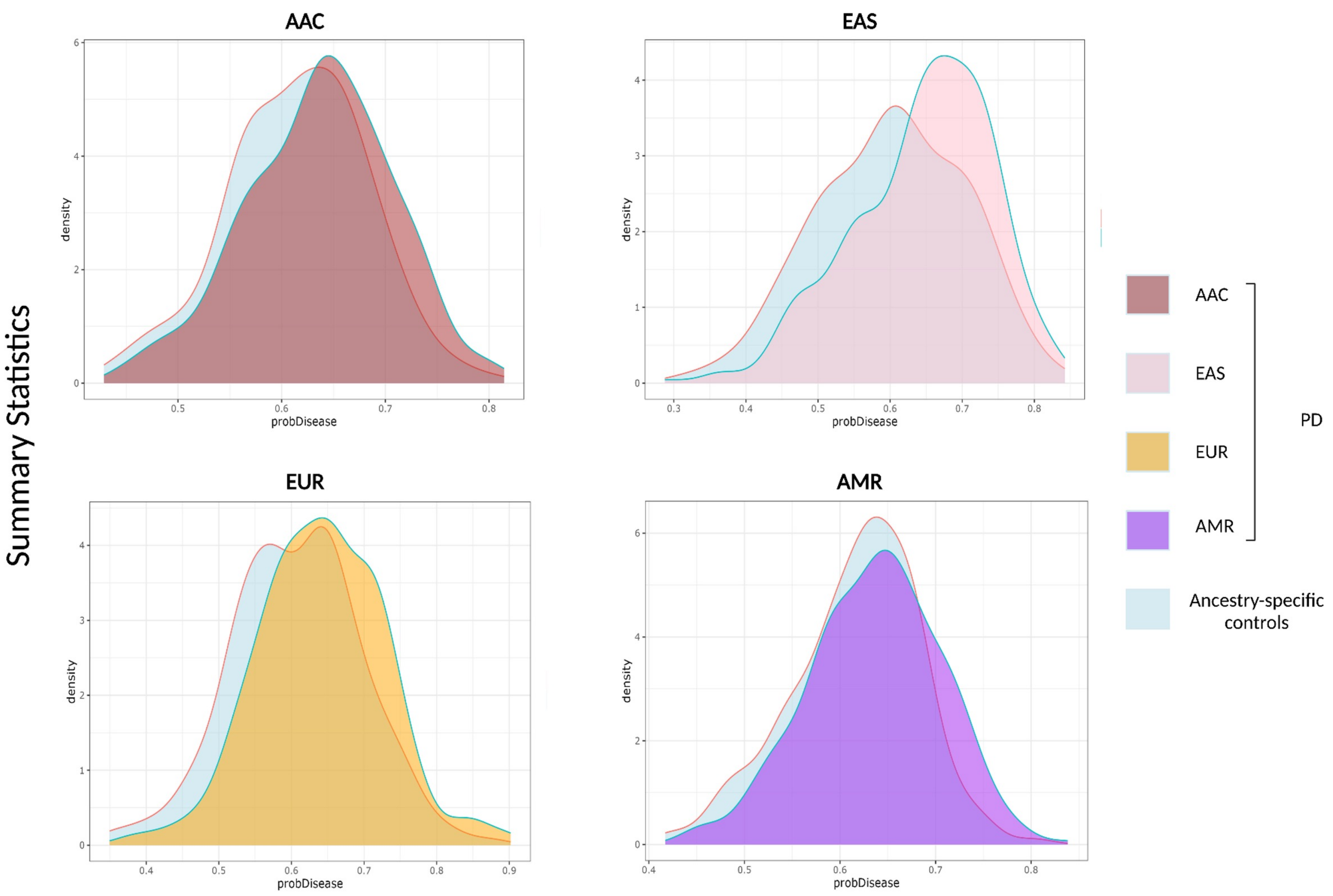

Supplementary Figure 4d: Disease probabilities in Latino/Admixed American(AMR) individual level data

The density plot illustrates disease probabilities in AAC individual-level data, with four panels representing the performance of population specific summary statistics (EUR, AMR, EAS, and AAC) weighted PRSes. Brown, pink, yellow, and purple curves represent PD cases (AAC, EAS, EUR, and AMR, respectively), while ancestry-specific controls are depicted in light blue.

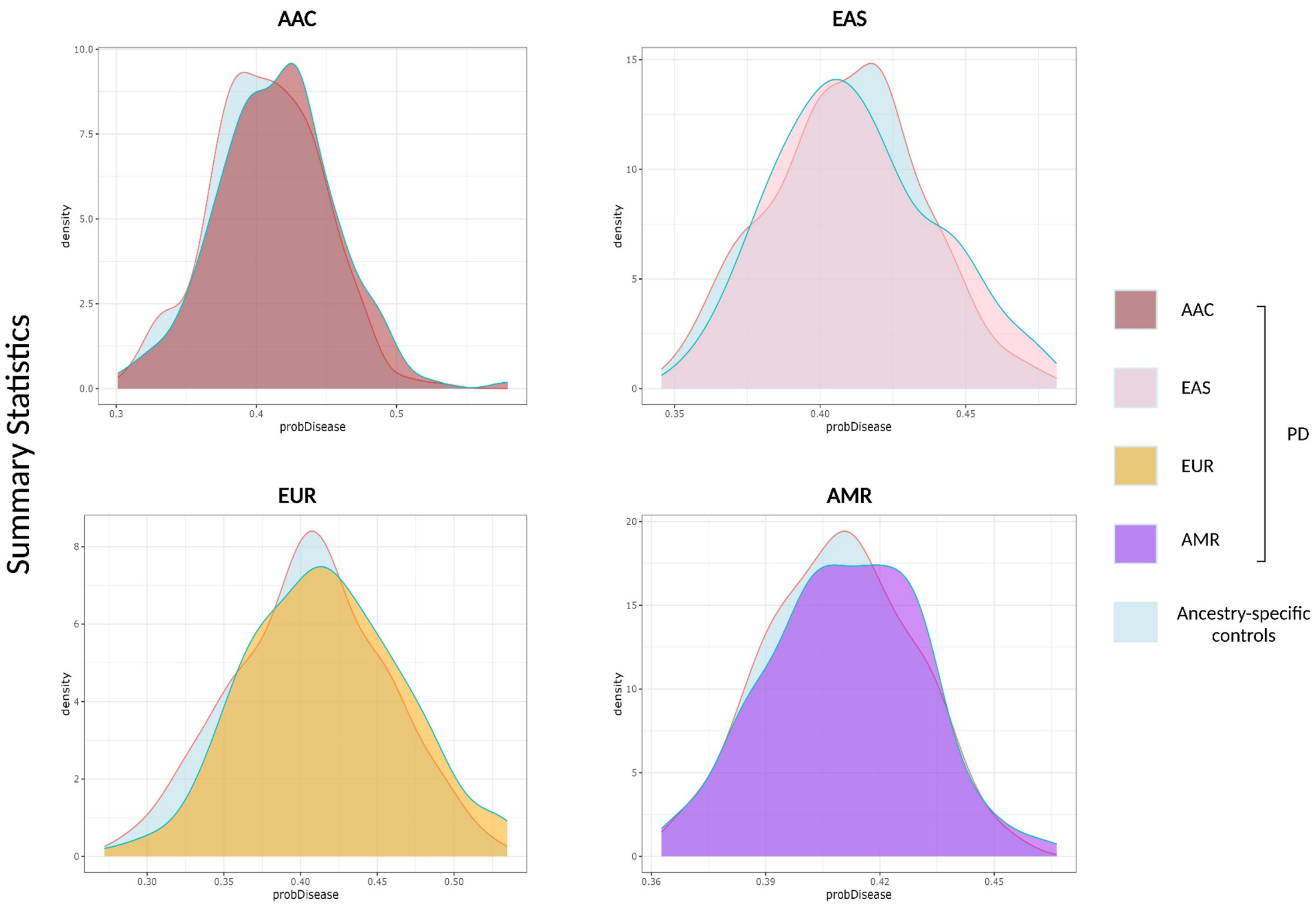

Supplementary Figure 4d: Disease probabilities in Central Asian (CAS) individual level data
The density plot illustrates disease probabilities in AAC individual-level data, with four panels representing the performance of population specific summary statistics (EUR, AMR, EAS, and AAC) weighted PRSes. Brown, pink, yellow, and purple curves represent PD cases (AAC, EAS, EUR, and AMR, respectively), while ancestry-specific controls are depicted in light blue.

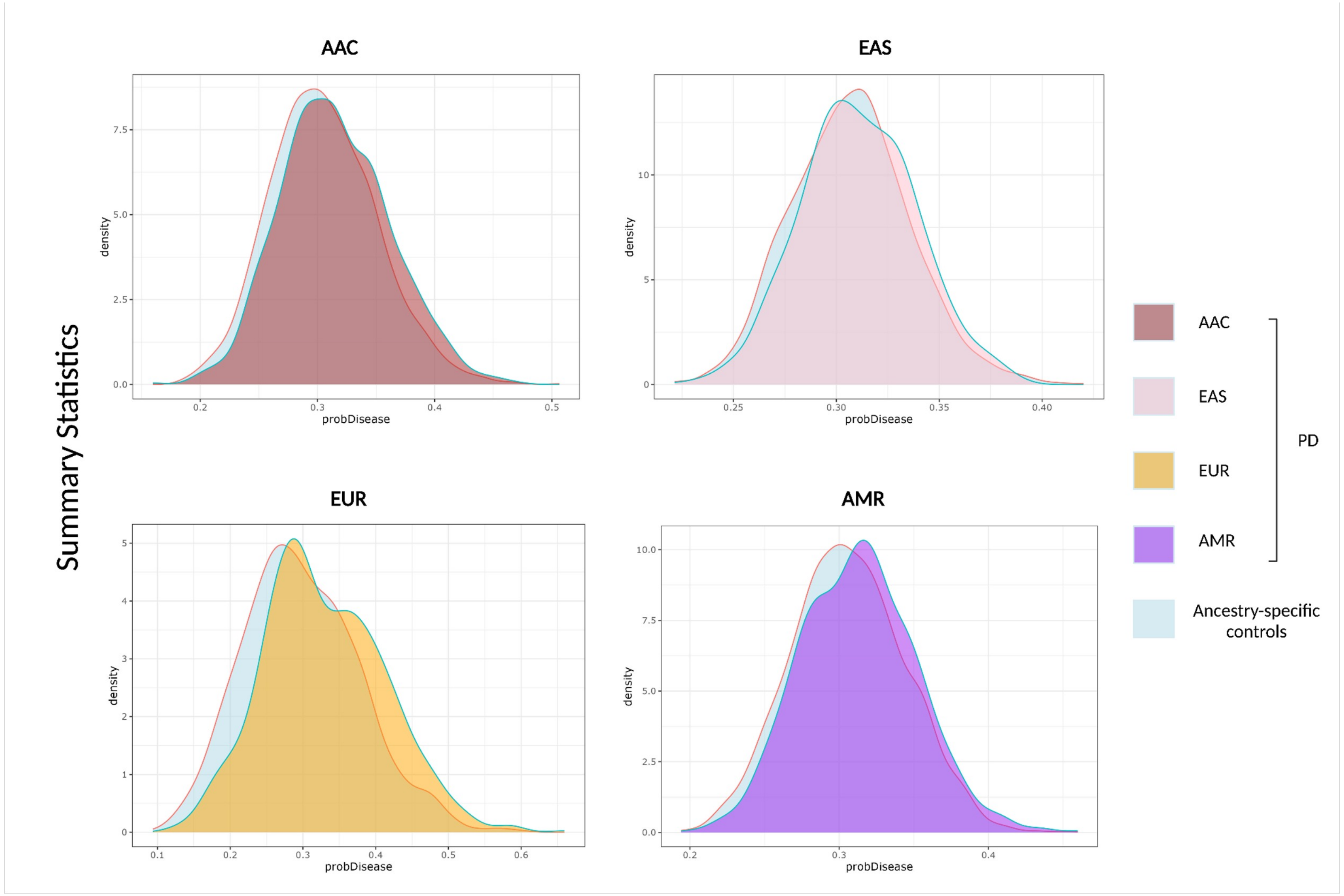

Supplementary Figure 4f: Disease probabilities in East Asian (EAS) individual level data
The density plot illustrates disease probabilities in AAC individual-level data, with four panels representing the performance of population specific summary statistics (EUR, AMR, EAS, and AAC) weighted PRSes. Brown, pink, yellow, and purple curves represent PD cases (AAC, EAS, EUR, and AMR, respectively), while ancestry-specific controls are depicted in light blue.

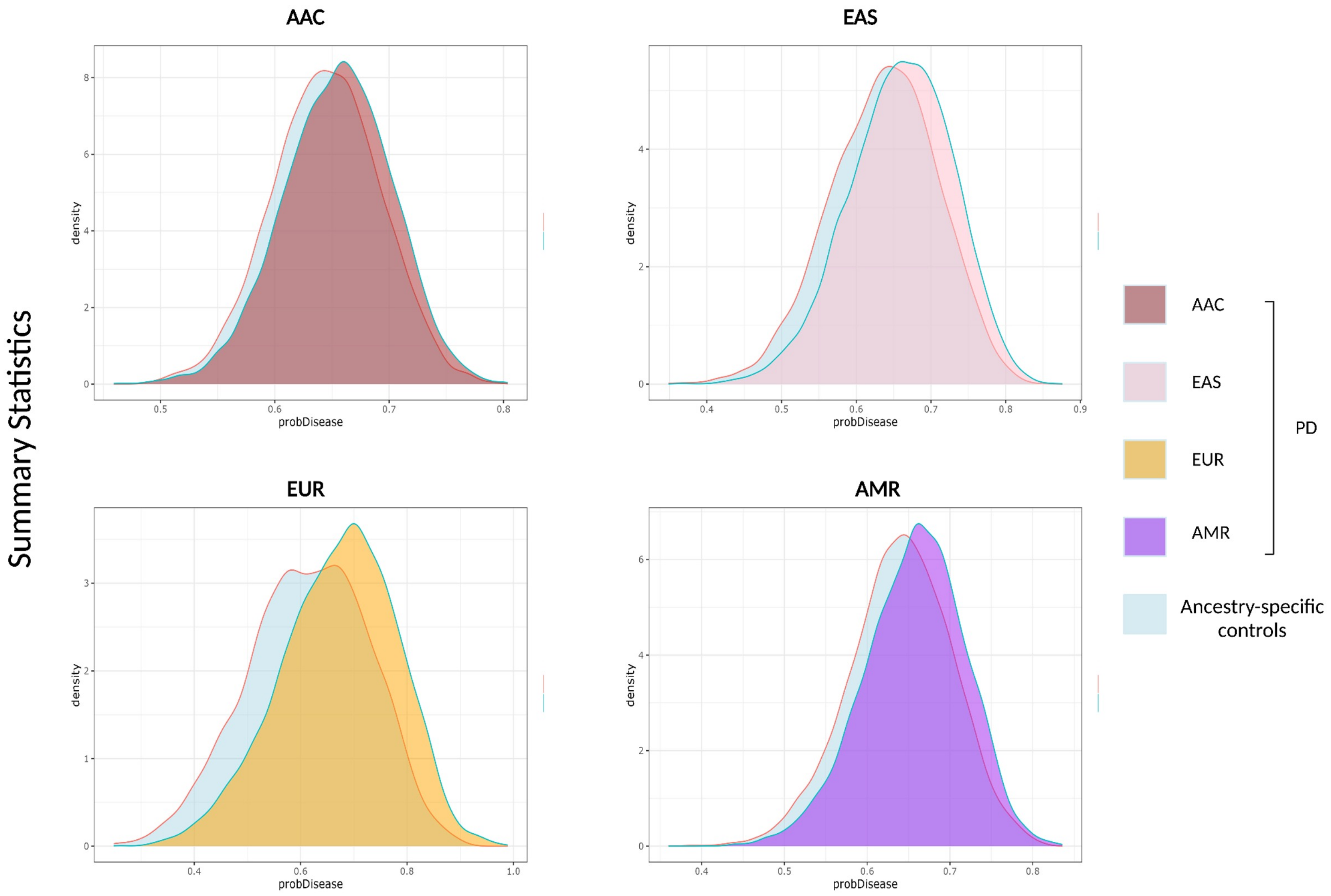

Supplementary Figure 4g: Disease probabilities in European (EUR) individual level data

The density plot illustrates disease probabilities in AAC individual-level data, with four panels representing the performance of population specific summary statistics (EUR, AMR, EAS, and AAC) weighted PRSes. Brown, pink, yellow, and purple curves represent PD cases (AAC, EAS, EUR, and AMR, respectively), while ancestry-specific controls are depicted in light blue.
